# Er:YAG Laser in QSP Modality for Treatment of Indirect Adhesive Restoration Build-Up: Surface Roughness Analysis and Morphology Assessment by Environmental Scanning Electron Microscopy (ESEM)

**DOI:** 10.3390/dj13050223

**Published:** 2025-05-21

**Authors:** Ilaria Giovannacci, Monica Mattarozzi, Fabrizio Moroni, Giuseppe Pedrazzi, Paolo Vescovi, Maria Careri

**Affiliations:** 1 Oral Medicine and Oral Laser Surgery Unit, Department of Medicine and Surgery, University of Parma, Via Gramsci 14, 43125 Parma, Italy; paolo.vescovi@unipr.it; 2 Department of Chemistry, Life Sciences and Environmental Sustainability, University of Parma, Parco Area delle Scienze 17/A, 43124 Parma, Italy; monica.mattarozzi@unipr.it (M.M.); maria.careri@unipr.it (M.C.); 3 Department of Engineering and Architecture (DISTI), University of Parma, Parco Area delle Scienze, 181/A, 43124 Parma, Italy; fabrizio.moroni@unipr.it; 4 Department of Medicine and Surgery, Unit of Neuroscience, Plesso Biotecnologico Integrato, University of Parma, 43125 Parma, Italy; giuseppe.pedrazzi@unipr.it; 5 Interdepartmental Center of Robust Statistics (Ro.S.A.), University of Parma, 43125 Parma, Italy

**Keywords:** adhesive dentistry, sandblasting, Er:YAG laser, roughness, ESEM

## Abstract

**Background/Objectives:** Sandblasting build-ups before applying the acid and adhesive significantly improves the bond strength. The aim of this study is to evaluate, for the first time, the effectiveness of an Er:YAG laser used in QSP mode to treat the surface of build-ups before the adhesive cementation sequence. **Methods:** This ex vivo study was conducted on 12 intact, undecayed extracted teeth kept hydrated in NaCl 0.9% solution. A cavity was created in the center and reconstructed with composite resin (build-up). Then, samples were prepared with burs and divided into three groups: control group G1, prepared only with burs; group G2, in which surfaces were treated with a sandblaster (2.5 bar, 10 mm from composite surface, aluminum oxide, 10 s); and group G3, treated using an Er:YAG laser (QSP modality, 1 W, 10 Hz, 100 mJ). The surface roughness of the build-ups was measured using a CCI MP-L digital optical profiler (Taylor Hobson, Leicester, UK), and surface morphology was studied using the Quanta™ 250 FEG (FEI, Hillsboro, OR, USA) ESEM instrument. **Results:** Regarding enamel, mean surface roughness in G1 vs. G2 was not statistically significant (*p* = 0.968); meanwhile, differences between the Er:YAG laser group (G3) and G1 or G2 were significant (G3 vs. G1 *p* < 0.001; G3 vs. G2 *p* < 0.001). Regarding dentin, G1 vs. G2 was significant (*p* = 0.021); differences between G3 and G1 or G2 were extremely significant (G3 vs. G1 *p* < 0.001; G3 vs. G2 *p* < 0.001). The same trend was detected for resin. **Conclusions:** An Er:YAG laser in QSP mode used on the build-up surface for indirect adhesive restorations is innovative and should be investigated with further studies. However, it seems extremely effective with increased roughness, the absence of a smear layer and characteristics potentially favorable for good adhesion for all substrates (enamel, dentin, resin).

## 1. Introduction

Modern dentistry has been characterized by the development and evolution of adhesive procedures. Adhesive systems have influenced the approach to restoring posterior teeth, modifying treatment plan considerations [1]. In particular, in large cavities with cusp coverage, it is clinically more favorable to use an adhesively cemented restoration as the first treatment choice. Veneziani defined an “adhesive indirect restoration” as a partial crown restoration made in composite or full ceramic, which has to be seated passively and adhesively cemented in a cavity characterized by specific attributes [1]. These preparations allow for greater preservation of healthy tissue compared to a metal-free full crown [2,3]. So, nowadays, adhesive indirect restoration is the new paradigm between restorative dentistry and prosthodontics. Indications for indirect adhesive restorations of posterior teeth are mainly esthetic purposes, bioeconomic principles and biomechanical strengthening of the remaining tooth structure [1,4]. Indirect adhesive restorations, regardless of material and shape, must be applied with an adhesive cementation technique using a rubber dam. The adhesive cementation process involves steps on the overlay and steps on the build-up. The steps on the overlay, which is generally sandblasted in the laboratory, depend on the material of the artifact itself; on the build-up, the traditional steps involve the application of 37% orthophosphoric acid and adhesive. However, it is now known in the literature that sandblasting the build-up before applying the acid and adhesive significantly improves the bond strength. In their review, D’Arcangelo et al. concluded that sandblasting treatment is the main factor responsible for improving the retentive properties of indirect composite restorations [5,6]. Erbium family lasers (erbium-doped: yttrium-aluminum-garnet (Er:YAG) and erbium-chromium; yttrium-scandium-gallium-garnet (Er,Cr:YSSG)) have been widely proposed for enamel surface conditioning in adhesive procedures. In particular, an Er:YAG laser emits radiation at a wavelength of 2.94 μm, while an Er,Cr:YSSG laser emits it at 2.78 μm, which water and hydroxyapatite can absorb. For this reason, they can be used as an etching adhesive in the treatment of tooth enamel [7]. Recently, a novel quantum square pulse (QSP) mode has been added to the range of treatment parameters of variable square pulse (VSP) Er:YAG dental lasers. Er:YAG QSP mode works by splitting a standard laser pulse of longer duration (approximately 600 μs) into a series of five super-short pulses (pulse quanta) that follow each other at an optimal effective rate (several kHz); this mode enables the delivery of laser energy with the same efficiency of short-duration pulses without sacrificing the precision provided by the long-duration pulses. This is because the duration of each of the pulse quanta (approximately 50 μs) is shorter than the rise time of the debris cloud, while the separation between the pulse quanta of approximately 85 μs is longer than the decay time of the debris cloud [8]. One of the major advantages of the QSP mode is that it significantly reduces the undesirable residual side effects of laser beam scattering and absorption in the debris cloud that forms immediately after the ablation of dental tissues. When a standard erbium laser beam is absorbed in the debris cloud, the ablation rate of dental tissue is lowered. Additionally, the scattering effect caused by the cloud leads to the spreading of the laser beam. Further, the laser-heated debris cloud is expected to contribute to the heating of the tooth as it falls back to the tooth surface [8,9]. By using QSP mode, the ablation cavities are sharp and well defined, with minimal thermal effects at the edges of the cavities [10]; Lukac et al. demonstrated that this mode is indicated as an ideal treatment modality for fast, high-quality cavity preparation [10,11]. In particular, the study of Lukac et al. is the only study present in the literature that investigates the morphology of the tooth after treatment with QSP mode, highlighting that the surfaces are free of smear layers and appear to have the high quality required for a high bond strength [9]. In this context, the aim of this preliminary study is to evaluate, for the first time, the effectiveness of an Er:YAG laser used in QSP mode to treat the surface of build-ups before the adhesive cementation sequence. In particular, this ex vivo study aims to characterize the surface morphology and roughness of build-ups treated with a QSP Er:YAG laser by environmental scanning electron microscopy (ESEM) and profilometry. In addition, these results are compared with those obtained from traditional sandblasting and from a control group prepared with burs without further surface treatment. The null hypothesis is that no substantial difference is found between treatment with a traditional sandblaster and an Er:YAG laser in QSP mode.

## 2. Materials and Methods

**Figure 1:**
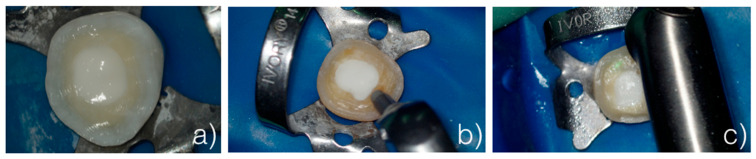
Sample from (**a**) control group (G1), (**b**) sandblaster group (G2), and (**c**) Er:YAG laser group (G3).

This study was performed ex vivo, on extracted human teeth. In particular, the samples were undecayed molars kept hydrated in 0.9% NaCl solution. A cavity was created in the center of each tooth, which was reconstructed using a composite resin (simulating a build-up). The build-up was then prepared with FG burs, simulating the preparation of a build-up, which is carried out in vivo for the execution of an indirect adhesive restoration with total cusp coverage (overlay). A total of 12 teeth were prepared in this way. Then, the samples were randomly divided into 3 groups (Figure 1):

− Control group (G1): The samples were only prepared with burs and not sandblasted (Figure 1a).
− Sandblaster group (G2): After preparation with burs, the surfaces were treated with an intraoral sandblaster (Microetcher CD, Kavo, Biberach, Germany), 2.5 bar pressure, approximately 10 mm from the surface—aluminum oxide (Al_2_O_3_, mean particle size 30 μm) powder—for 10 s (Figure 1b).
− Er:YAG laser group (G3): After preparation with burs, the surfaces were treated with an Er:YAG laser (Fotona LighWalker^®^, Ljubljana, Slovenia), in QSP mode—power: 1 W; frequency: 10 Hz; energy: 100 mJ (Figure 1c).

Two samples from each group were randomly chosen for surface morphology analysis. For surface roughness analysis, 2 other samples were randomly selected from each group.

### 2.1. Surface Roughness Analysis

**Figure 2:**
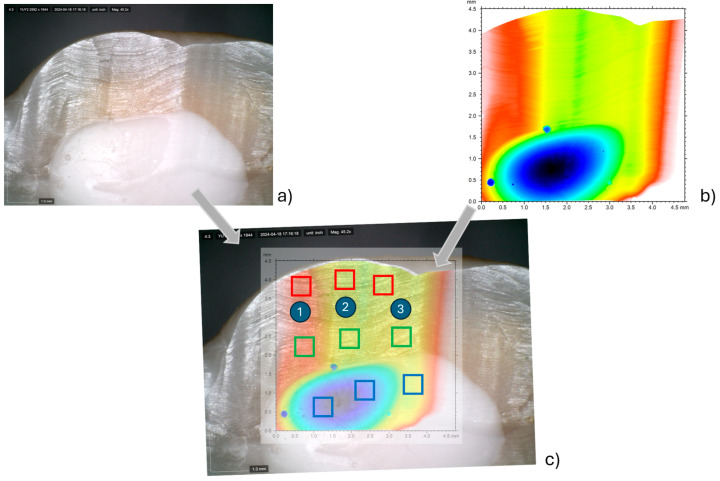
Example of morphology map acquired for the control specimen (**a**); digital camera acquisition for the untreated specimen (**b**); identification of the three sub-areas for each portion of the surface (**c**).

Surface roughness was measured using a CCI MP-L digital optical profiler (Taylor Hobson, Leicester, UK). For each treatment considered, an area of 4.7 mm × 4.7 mm was scanned with a resolution of 3.4 μm on the XY plane and 5 nm along the vertical (Z) axis. An example of the resulting map is shown in Figure 2a. The sample surface was then acquired using a 5 MPx Dinolite digital camera (Almere, The Netherlands), as shown, for example, in Figure 2b, to clearly identify the three corresponding portions of the specimen, i.e., enamel, dentin and resin, in the morphological map. Subsequently, the two acquisitions (from the profiler and from the digital camera) were overlapped, and for each portion of the surface, three smaller sub-areas of 0.4 mm × 0.4 mm were extrapolated (Figure 2c). Finally, for each sub-area, the average surface roughness, Sa, according to ISO 25178-2:2012 [12] was computed.

#### Statistical Evaluation

The data analysis was performed using the open-source statistical package Jamovi v. 2.6.44 (www.jamovi.org, accessed on 31 March 2025), which is based on the well-known and widely used R statistical environment (https://cran.r-project.org/, accessed on 31 March 2025). For the descriptive analysis of continuous variables, the main measures of central tendency and dispersion were calculated, including mean, median, standard deviation, interquartile range, minimum, maximum, and range. Where relevant, standard errors and their 95% confidence intervals were also reported. For the graphical part, boxplots and interval plots were used, which allowed for the immediate visualization of central tendency (median and mean) and comparison between different data groups. For the inferential analysis of continuous variables, parametric tests such as Student’s *t*-test, ANOVA and repeated measures ANOVA were used, after verifying the assumptions of homogeneity and normality. The Shapiro–Wilk test was used to assess normality, while Levene’s test was used to assess the homogeneity of variances. Results were considered statistically significant when the *p*-value was less than 5% (*p* < 0.05). Post hoc analysis was performed using Tukey’s HSD test.

### 2.2. Surface Morphology Analysis by ESEM

**Figure 3:**
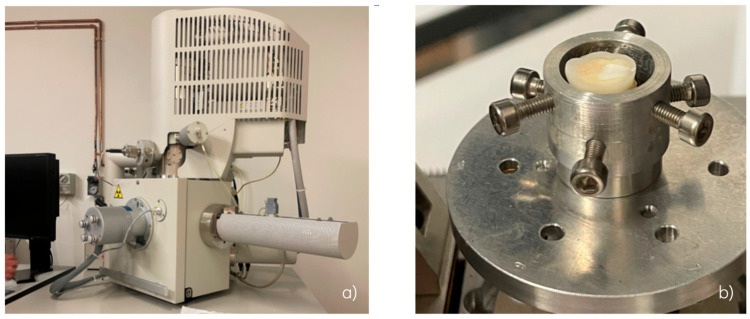
(**a**) Environmental scanning electron microscope Quanta™ 250 FEG (FEI, Hillsboro, OR, USA). (**b**) Sample mounted on a home-made stainless-steel support for ESEM analysis.

The Quanta™ 250 FEG (FEI, Hillsboro, OR, USA) ESEM instrument was used for morphological analysis (Figure 3a). The secondary electron signal was recorded for topographic contrast by using a Large-Field Detector (LFD), using a low kV 500 μm Pressure-Limiting Aperture (PLA) cone. The PLA cone profile extends down to 3 mm. The tooth was mounted without any previous preparation on a home-made stainless-steel support in order to keep the specimen vertically oriented, and then transferred into the microscope chamber (Figure 3b). The microscope operated in low-vacuum mode at 100 Pa with an accelerating voltage of 5 kV, a spot size of 2.5, a final lens aperture of 30 μm and a working distance of 8 mm. For each sample, a 15 × 15 navigation montage was preliminarily acquired to explore its structure; then, the obtained image was used as reference for further investigation at different magnifications.

## 3. Results

### 3.1. Results from Surface Roughness Analysis

**Figure 4:**
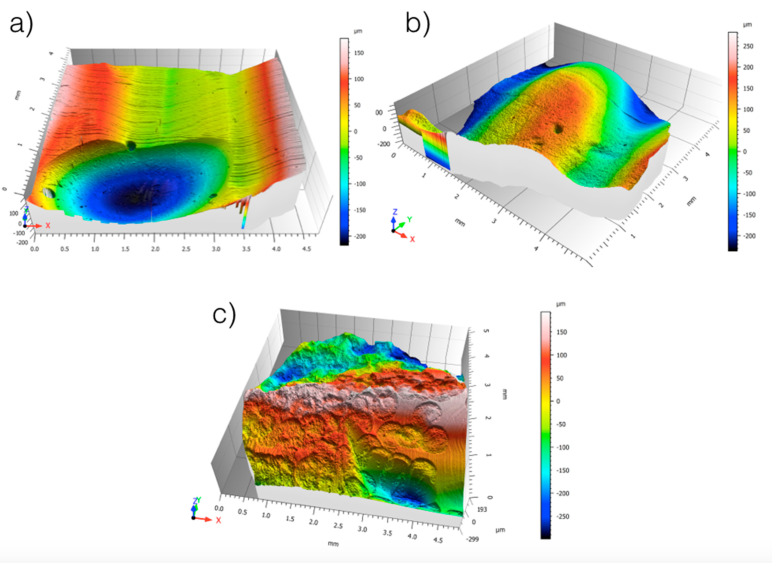
Morphology map acquired for (**a**) control, (**b**) sandblaster and (**c**) Er:YAG laser samples.

**Figure 5:**
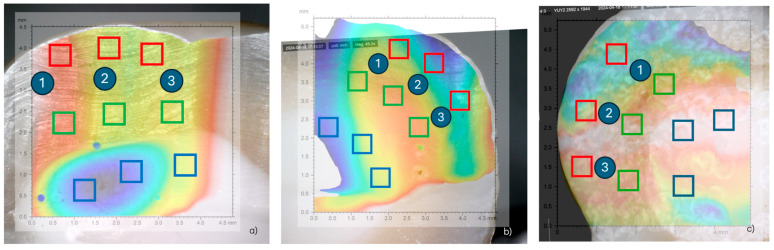
Locations of the considered sub-areas for (**a**) control, (**b**) sandblaster and (**c**) Er:YAG laser samples.

Figure 4 shows the morphology maps acquired for the control, sandblasted and laser-treated specimens. The spots created by the laser pulses can be clearly identified (Figure 4c). The sub-areas in which the Sa values were calculated are represented in Figure 5. The obtained results are summarized in Table 1. One-way ANOVA returned statistically significant results for enamel (*p* = 0.002), dentin (*p* < 0.001) and resin (*p* < 0.001) (Table 2).

**Figure 6:**
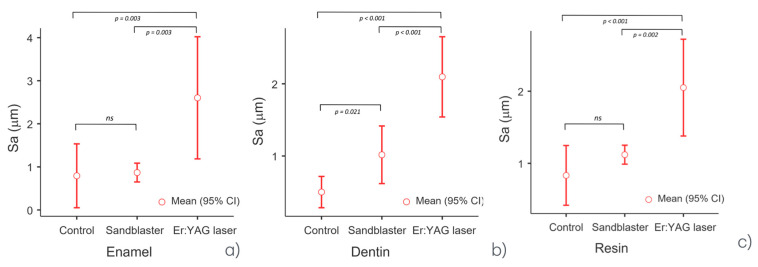
Graph showing comparison between groups regarding (**a**) enamel, (**b**) dentin and (**c**) resin.

**Figure 7:**
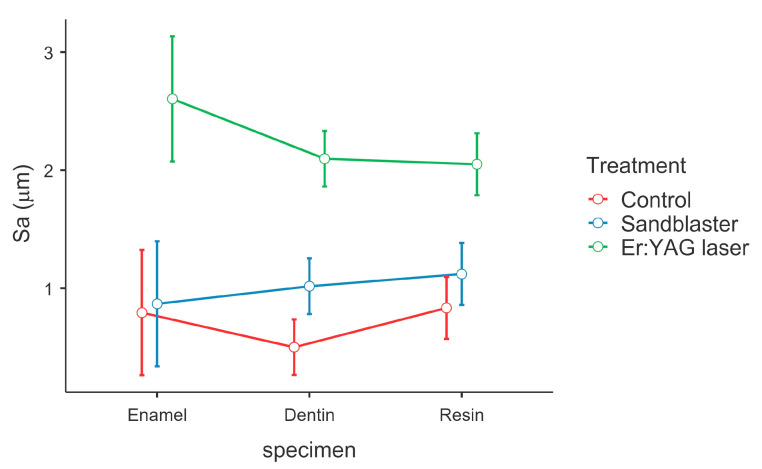
Graph showing ANOVA for repeated measures.

For each area analyzed, Tukey’s post hoc test was performed in order to identify which comparisons between groups were significant. In particular, regarding enamel, the control group (G1) versus the sandblaster group (G2) was not statistically significant (*p* = 0.968); meanwhile, the differences between the Er:YAG laser group (G3) and G1 or G2 were statistically significant (G3 vs. G1 *p* < 0.001; G3 vs. G2 *p* < 0.001). Regarding dentin, the control group (G1) versus the sandblaster group (G2) was significant (*p* = 0.021); the differences between the Er:YAG laser group (G3) and G1 or G2 were extremely significant (G3 vs. G1 *p* < 0.001; G3 vs. G2 *p* < 0.001). The same trend was detected for resin. Comparison between groups showed that G3 versus G2 and G1 was significant (*p* = 0.002 and *p* < 0.001, respectively), while G2 versus G1 was not statistically different. The comparisons between groups for enamel, dentin and resin are represented in Figure 6 and Table 3. Furthermore, an ANOVA test for repeated measures was performed in order to obtain an overview of the individual treatments and surfaces. The post hoc test for repeated measures showed that there were no statistically significant differences between enamel, dentin and resin; meanwhile, a statistically significant difference was confirmed between G1 and G3, and G2 and G3, but not between G2 and G1 (Figure 7).

### 3.2. Results from Surface Morphology Using ESEM

**Figure 8:**
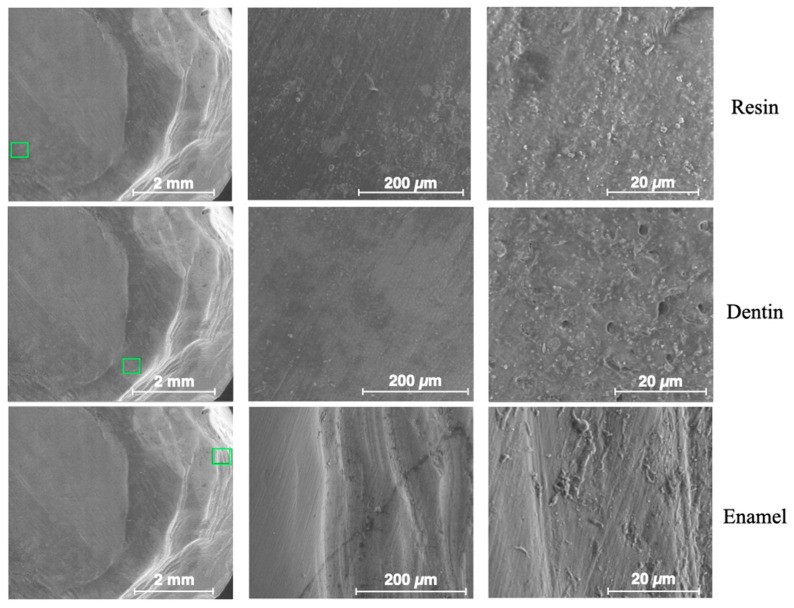
ESEM images from the control group—G1. The green frame indicates the point on the sample from which magnified images were obtained.

**Figure 9:**
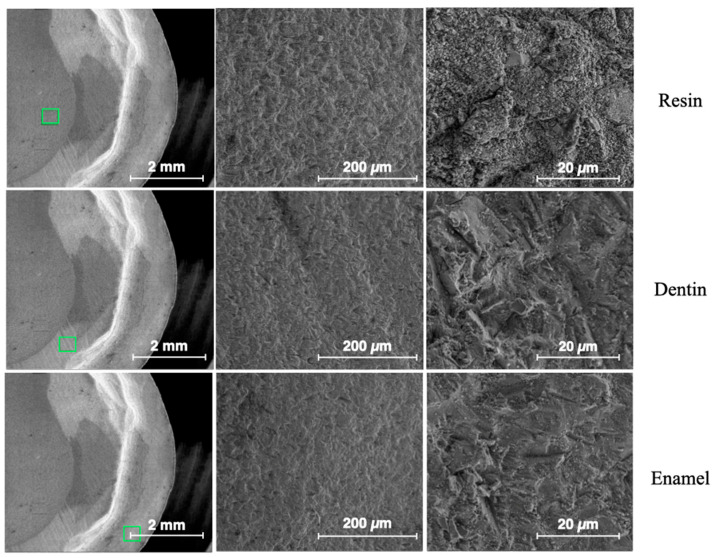
ESEM images from the sandblaster group—G2. The green frame indicates the point on the sample from which magnified images were obtained.

**Figure 10:**
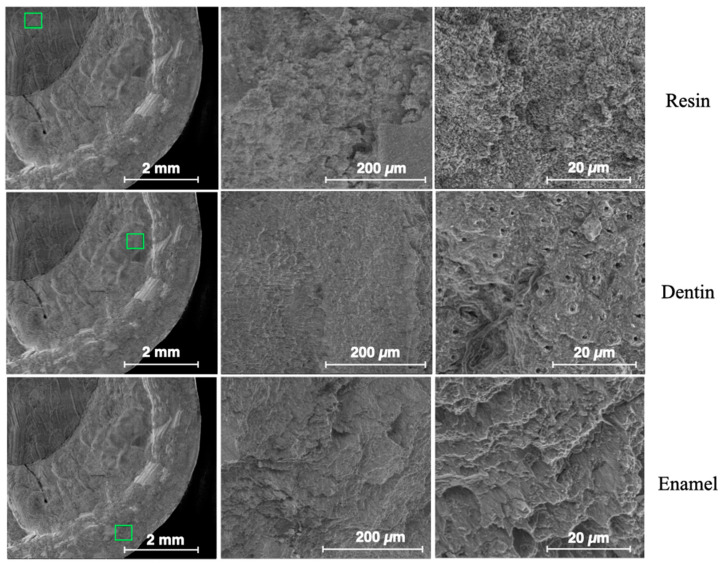
ESEM images from the laser group—G3. The green frame indicates the point on the sample from which magnified images were obtained.

**Figure 11:**
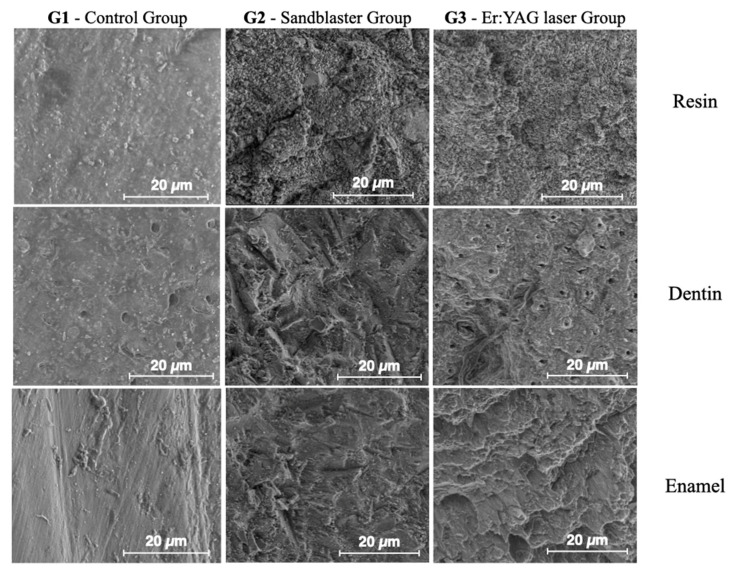
Comparison panel between each group for enamel, dentin and composite resin.

**Figure 12:**
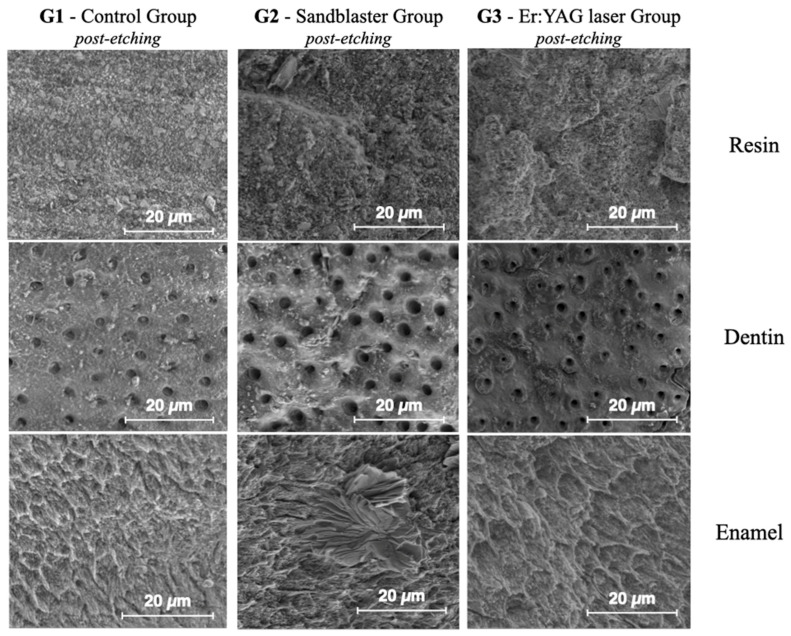
Comparison panel between each group for enamel, dentin and composite after application of 37% orthophosphoric acid.

Figure 8 shows ESEM micrographs at different magnifications for the enamel, dentin and resin in the control sample. The surfaces appear very flat, especially for the enamel. Figure 9 shows typical ESEM images with different magnifications of the enamel, dentin and resin in the sandblasted sample. Higher roughness is evident compared to the control group, but there is a notable smear layer, especially in the dentin. Prisms start to appear in the enamel, but covered by a notable smear layer. Figure 10 depicts typical ESEM images with different magnifications of the enamel, dentin and resin in the laser sample. Roughness is evident in all three substrates but in the total absence of a smear layer. Tubules and a fishbone appearance are visible in the dentin. There are clearly visible prisms in the enamel, almost as if there has been an etching action without having applied the orthophosphoric acid yet. Figure 11 shows a comparison panel between each group for enamel, dentin and composite. The surfaces treated with the sandblaster and laser (G2 and G3) appear rougher than the control ones (G1), but it is noteworthy that in the laser sample, there is no smear layer, tubules are visible, and the enamel has organized prisms (similar to an etching action). ESEM allowed the same sample to be re-examined after it had undergone etching through the application of 37% orthophosphoric acid for 15 s on the enamel and 30 s on the dentin and resin. As shown in Figure 12, the resin presents greater roughness in the sample treated with the laser followed by etching; the roughness is both inside the laser spot and between spots. As for dentin, in the laser sample, there is less of a smear layer, and the tubules are open but smaller in diameter. Only in the laser sample is the peritubular dentin in relief, making retention potentially favorable. In the enamel, open prisms are visible in all samples, but the laser combined with etching resulted in less of a smear layer and prisms of a more regular shape (honeycomb appearance).

## 4. Discussion and Conclusions

Modern restorative dentistry is essentially adhesive. The goal of adhesive dentistry is the maximum preservation of healthy tissue (not only dental but also pulp and periodontal). With this approach, indirect adhesive restorations are indicated in large cavities associated with cusp coverage with no or reduced amounts of cervical enamel. To guarantee this, retention is no longer given by mechanical but adhesive concepts, linked to the treatment of the surfaces of both the overlay and the tooth (build-up). In addition to the various etching and adhesive application procedures, for many years, sandblasting of surfaces has been demonstrated to increase the adhesion effectiveness. The increase in the surface intended for adhesion, but also in the roughness and surface energy, constitutes the basic mechanism through which sandblasting takes effect. According to some authors, its contaminant-cleansing action is also not negligible [1,2]. While the overlay is usually sandblasted in the laboratory, the build-up is sandblasted under a rubber dam using an intraoral sandblaster before applying the etchant. The aim of this preliminary study was to evaluate the effectiveness of an Er:YAG laser in QSP mode to treat the surface of build-ups before the application of the etchant. The application of the Er:YAG laser was compared with the action of an intraoral sandblaster on the build-up. In QSP mode, a longer laser pulse is split, or “quantized”, into multiple short pulses that follow each other at an optimal rate. This allows the QSP mode to deliver precise, fine-tuned pulses with the efficiency of long-duration laser pulses without sacrificing the accuracy of shorter pulses [7]. Less scattering means better effectiveness, more precision and reduced thermal effect. Lukac et al. studied the effectiveness of QSP mode on dentin and enamel. The surfaces treated with QSP appeared to have a high quality that was suitable for a high bond strength, as well as being free of smear layers. In particular, the dentin appeared clean, regular and flat with well-opened tubules, with no difference between the intertubular and peritubular dentin. The enamel appeared clean and homogeneous with well-defined microroughness [8]. To the best of our knowledge, there is a lack of specific studies on the use of lasers in the treatment of the build-up of indirect restorations. However, the use of Er:YAG lasers for the treatment of enamel and dentin in other situations such as cavity preparation, orthodontic bracket bonding and filling removal has been widely investigated. Regarding the parameters used for the treatment of the build-up in the present study, they agree with what emerges from the literature. In 2022, Labunet et al. concluded that it is recommended to use an Er:YAG laser for enamel treatment before bonding the brackets, paying attention to microcracks and thermal damage [10]. In another systematic review and meta-analysis, Jiang et al. demonstrated that an Er:YAG laser is useful for the treatment of enamel for bonding brackets, but only when using specific parameters. In this study, the optimum ranges of the laser parameters were found to be 1~1.5 W, 100~120 mJ, 10~20 Hz and a wavelength of 2.94 μm for an Er:YAG laser [7]. Wang et al. performed research focused on laser treatment on dentin. The authors demonstrated that treatment using an Er:YAG laser is associated with opened dentinal tubules, protrusion of peritubular dentin and the absence of a smear layer, but only when using delicate parameters. In particular, the authors demonstrated an optimum range of energy from 50 to 200 mJ and frequency from 5 to 20 Hz. Over these parameters, you can see damage on dentin [11]. On the contrary, in their study, Akhoundi et al. demonstrated that Er:YAG laser treatment was associated with damage in enamel structures. However, paying attention to the parameters used, they are much higher than those emerging from the literature: 2 W, 200 mJ, 10 Hz, not QSP mode [13]. In the study presented in this work, 1 W of power, 100 mJ of energy and 10 Hz were used. In particular, using the QSP mode, 100 mJ of energy can be divided into five pulselets of 20 mJ, with an even more delicate effect, with the same total energy [13]. The potential of ESEM was exploited for the microstructure characterization of resin, dentin and enamel surfaces. In fact, ESEM has extended the application field of conventional scanning electron microscopy to the direct analysis of non-conductive and hydrated samples, without prior sample preparation. A noteworthy advantage of this technique is the analysis of untreated samples in their native state, with this preventing the introduction of artifacts during sample preparation and opening the possibility to perform specific experiments in sequential steps. This last aspect is of paramount importance for dynamic experiments, where the effect of a particular treatment needs to be studied on the same sample. For these reasons, ESEM may help to extend existing knowledge of the mechanisms of processes used in the clinical practice of dentistry. In particular, it is possible to analyze dental tissues in their native form, without the need for conductive coatings, and it is possible to analyze the same sample before and after phases such as etching [14,15,16,17]. Optimal substrates for adhesion emerged from the analysis of the morphology of the surfaces using ESEM. In particular, in the samples treated with the Er:YAG laser, the build-up composite was rougher without a smear layer; the enamel exhibited evident and well-organized prisms with a honeycomb appearance, almost as if it had already been etched. The dentin appeared free of smear layers and powder particles with open tubules and a fishbone appearance. The surfaces of the control sample were extremely flat, and those treated with a traditional sandblaster were rougher than the control but with a notable smear layer covering the tubules and prisms. Surface roughness was measured with a laser profilometer and the results were statistically processed. In the enamel, in the dentin and in the composite, the roughness values obtained with the laser were significantly higher than those obtained with the sandblaster and for the control. In particular, the greatest difference was obtained in the enamel, and this is a very important result because in the field of indirect adhesive restorations, the residual dental enamel is the main structure on which the adhesion is based. Dentin is generally only minimally represented in the build-up. In conclusion, laser treatment of the build-up surface before etching is associated with an increase in roughness in all substrates (enamel, dentin, resin), making micro-mechanical retention potentially favorable. The surfaces treated with the laser have less of a smear layer than those treated with a sandblaster and with burs only, and the combination of laser and etching by orthophosphoric acid produces a very clean honeycomb texture in the enamel prisms, very favorable for the adhesive cementation of indirect restorations. However, these are preliminary results, and they should be followed up by the study of the bond strength. The study of the bond strength will be fundamental to understanding whether the data emerging from this preliminary study can also be translated into a better clinical efficacy of adhesive cementation. The surface roughness in the absence of dust and a smear layer and the microscopic morphological aspect seem to be perfect for adhesion. Nevertheless, future study steps will be focused on the study of the bond strength and on the study of the clinical efficacy. In particular, it will be desirable to evaluate the efficacy of the treatment proposed in this study, through the in vivo evaluation of variables such as the incidence of secondary decay, microcracks in the build-up, chipping of the overlays and post-cementation hypersensitivity.

## Figures and Tables

**Table 1:** Descriptive results obtained for each group.

Descriptives						
	Treatment	Mean	Median	SD	Minimum	Maximum
Enamel	Control (G1)	0.793	0.687	0.299	0.562	1.13
	Sandblaster (G2)	0.868	0.840	0.088	0.797	0.966
	Er:YAG laser (G3)	2.603	2.390	0.571	2.17	3.25
Dentin	Control (G1)	0.500	0.533	0.087	0.401	0.566
	Sandblaster (G2)	1.017	0.950	0.161	0.9	1.2
	Er:YAG laser (G3)	2.097	2.050	0.224	1.9	2.34
Resin	Control (G1)	0.833	0.887	0.167	0.646	0.966
	Sandblaster (G2)	1.120	1.100	0.053	1.08	1.18
	Er:YAG laser (G3)	2.050	2.050	0.270	1.78	2.32

**Table 2:** One-way ANOVA test obtained for enamel, dentin and resin.

One-Way ANOVA					
	F	df1	df2	ω²	*p*
Enamel	22.3	2	6	0.826	0.002
Dentin	71.6	2	6	0.94	<0.001
Resin	35.2	2	6	0.884	<0.001

**Table 3:** Tukey’s post hoc test between groups for enamel, dentin and resin.

Tukey Post Hoc Test—Enamel		
Control—sandblaster:	mean difference = −0.07	*p*-value = 0.968
Control—Er:YAG laser:	mean difference = −1.81	***p*-value = 0.003**
Sandblaster—Er:YAG laser	mean difference = −1.74	***p*-value = 0.003**
Tukey’s post hoc test—dentin		
Control—sandblaster:	mean difference = −0.52	***p*-value = 0.021**
Control—Er:YAG laser:	mean difference = −1.60	***p*-value < 0.001**
Sandblaster—Er:YAG laser	mean difference = −1.08	***p*-value < 0.001**
Tukey’s post hoc test—resin		
Control—sandblaster:	mean difference = −0.29	*p*-value = 0.221
Control—Er:YAG laser:	mean difference = −1.60	***p*-value < 0.001**
Sandblaster—Er:YAG laser	mean difference = −1.08	***p*-value = 0.002**

## Data Availability

The original contributions presented in this study are included in the article. Further inquiries can be directed to the corresponding author.

## Abbreviations

The following abbreviations are used in this manuscript:

QSP	Quantum square pulse
ESEM	Environmental scanning electron microscopy
Er:YAG	Erbium-doped: yttrium-aluminum-garnet
Er,Cr:YSSG	Erbium-chromium; yttrium-scandium-gallium-garnet
VSP	Variable Square Pulse
G1, G2, G3	Groups 1, 2 and 3
ANOVA	Analysis of variance
PLA	Pressure-Limiting Aperture
LFD	Large-Field Detector
